# Analysis of the Cutting Abilities of the Multilayer Grinding Wheels—Case of Ti-6Al-4V Alloy Grinding

**DOI:** 10.3390/ma15010022

**Published:** 2021-12-21

**Authors:** Dariusz Lipiński, Kamil Banaszek, Łukasz Rypina

**Affiliations:** 1 Faculty of Mechanical Engineering, Koszalin University of Technology, 75-620 Koszalin, Poland; lukasz.rypina@tu.koszalin.pl; 2 Doctoral School, Koszalin University of Technology, 75-453 Koszalin, Poland

**Keywords:** grinding, Ti-6Al-4V, abrasive aggregates, multilayer tool, bootstrap method, surface roughness

## Abstract

This paper presents an effectiveness analysis of the grinding process with the use of a new multi-layer abrasive tool. The designed abrasive tool consists of external layers with a conventional structure, whose task is to decrease the grinding wheel load and ensure high grinding volumetric efficiency. The inner layer of the grinding wheel contains a 30% addition of abrasive aggregates. The task of the inner layer is to provide lower roughness of the machined surface. The aim of the research presented in this paper was to evaluate the topography of the designed abrasive tool and to analyze the middle layer properties influencing the machined surface roughness. The differentiation of the active surface features of the abrasive tool was determined for the conventional layer and the layer with the addition of abrasive aggregates. The machining potential of the layers was also determined using the Shos parameter. The surface topography of Ti-6Al-4V alloys ground with the use of a multi-layer wheel and a conventional grinding wheel was analyzed. With the application of the bootstrap hypothesis, the set of roughness parameters differentiating the topography of ground surfaces was determined.

## 1. Introduction

The continuous development of technology implies the creation of new engineering materials, the properties of which allow for the construction of machines with a greater efficiency. An example is the Ti-6Al-4V alloy, which is a mixture of titanium in α + β phases. This alloy is characterized by a high strength to weight ratio, lower density than steel, the ability to maintain high mechanical strength at elevated temperatures, low thermal conductivity, and corrosion resistance [1]. This material is used, among others, in the chemical, biomedical, and aviation industries [1,2]. The properties of the alloy have led to increased machining difficulties. The low thermal conductivity of titanium results in a high concentration of the temperature field around the cutting zone [3]. This leads to thermal damage and geometric inaccuracies of the surface layer of the workpieces [4].

The high chemical activity of the Ti-6Al-4V alloy with poorly selected machining parameters can lead to strong bonding and welding between the active surface of the grinding wheel and the machined surface [5]. It has also been observed that a high chemical activity causes the formation of oxides, which act on the cutting blades like abrasive micro-grains [6]. Titanium and its alloys react with the abrasive grains to deposit the ground material on the cutting edges. Re-contact of such abrasive grains with the workpiece may result in the redeposition of the workpiece on the ground surface, which worsens the surface roughness after machining [7]. Additionally, due to the phenomenon of clogging the active surface of the grinding wheel with waste products of the grinding process, the grinding wheel loses its ability to self-sharpen, resulting in an increase in grinding forces and temperature in the machining zone [8].

Research on the grinding process of titanium alloys shows that the use of super abrasive grinding wheels (with cubic boron nitride (cBN) or diamond grains) ensures higher durability of the tools due to the higher strength and better thermal conductivity of their grains [5]. However, this requires the use of high-pressure cooling [9], which in turn increases the cost of the process related to the storage and filtration of the coolant and the disposal of liquid waste [10]. In addition, the cost of super abrasive wheels and the time and costs associated with the process of renewing their active surface is significantly higher than in the case of conventional grinding wheels with abrasive grains of aluminium oxide (Al_2_O_3_) or silicon carbide (SiC) [11]. However, abrasive grains made of Al_2_O_3_ or SiC have a lower thermal conductivity than grains made of cBN or diamond, so heat dissipation from the grinding zone is more difficult [12].

Despite the greater efficiency of the grinding process of titanium alloys with super abrasive tools, their wide and practical application in this process is still difficult, mainly due to the high cost of production [11]. This has led to an increase in research on the use of modified conventional abrasive tools to increase the efficiency of the grinding process of difficult-to-machine materials. Kacalak et al. [13] used an aggregate grinding wheel with alumina grains, the use of which increased the efficiency of the of Ti-6Al-4V alloy machining. The applied tool had an admixture of 30% abrasive aggregates to the conventional Al_2_O_3_ grains. During the grinding of the Ti-6Al-4V alloy, an improvement in the grinding process was proven compared to the conventional tools: (i) increased material removal efficiency; (ii) reduction of side ridges; (iii) reduction of grinding forces, and (iv) reduction of the Sa roughness parameter of the ground surface. The effectiveness of the material removal process with the use of abrasive aggregates could be a result of increasing the abrasives cutting edge length [14].

An important factor influencing grinding process efficiency is the grinding wheel’s ability to self-sharpen [15]. As a result of the abrasion wear of abrasive grains, as well as the adhesion of the workpiece material to the abrasive grains, the contact surface of the grinding wheel and the workpiece increases. This increases the thermal and mechanical interactions in the machining zone. The ability to self-sharpen (chipping the cutting edges or pulling out the abrasive grains) allows for the renewal of the grinding wheel‘s active surface. Research on the wear processes of abrasive aggregates indicates their high micro-sharpening ability [16,17]. This leads to an acceleration of its dimensional and shape wear, and thus to a reduction of the grinding wheel tool life. Increasing the durability of abrasive tools is possible by changing the loads on their zones, as a result of the appropriate shaping the active surface, with appropriately selected characteristics. Nadolny et al. [18,19] showed that the use of layered grinding wheels with appropriately selected characteristics reduced the grinding power and increased tool life compared with conventional grinding wheels.

Based on the literature analysis, a special abrasive tool was designed for efficient grinding of the Ti-6Al-4V alloy. The tool consisted of external layers with a conventional structure and an internal layer with 30% addition of abrasive aggregates. The task of the inner layer is to provide lower roughness of the machined surface. The assumption of placing the layer with abrasive aggregates as the middle layer of the tool was to use its machining potential while minimizing its load. The task of a specially shaped outer layer (with a conventional structure and larger size of abrasive grains) is to ensure high volumetric efficiency. The purpose of introducing the chamfer in the outer layer was to balance the load on the outer layer.

In this paper, an analysis of the surface topography of newly designed grinding wheels is presented. Potential areas of contact of individual layers of the grinding wheel with the workpiece are assessed. Their cutting ability was determined using the Shos parameter, which takes into account the directionality of the cutting edges of the analyzed areas, their sharpness, and elevation. An experimental study was carried out by grinding the Ti-6Al-4V titanium alloy with the use of a conventional grinding wheel and a multi-layer grinding wheel. The topography of the ground surface was assessed with the use of 33 roughness parameters. The significance of changes in the mean values of roughness parameters was performed with the use of a bootstrap test. A comparative analysis of the roughness parameters of machined surfaces with the use of a conventional grinding wheel and a multi-layer grinding wheel was performed.

## 2. Materials and Methods

**Figure 1:**
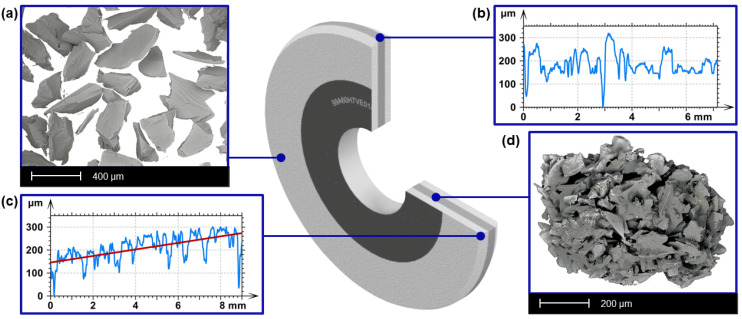
The structure and the geometrical layers properties of the multilayer grinding tool: (**a**) SEM image of a conventional grains in the external layers, (**b**) the profile of the internal cylindrical layer, (**c**) profile of the external conical layer, and (**d**) SEM image of an abrasive aggregate (the admixture to the conventional grains).

In order to improve the grinding process efficiency, a new multi-layer tool was designed (Figure 1). It consists of three layers. Externally it has a conventional composition and internally it consists of conventional grains and an admixture of the grinding aggregates. The abrasive grains in the outer layer are sized 60 according to the FEPA (Federation of European Producers of Abrasives) standard, the grains in the inner layer are sized F120, and the abrasive aggregates are F240. The edges of the outer layers were chamfered with a length of 9 mm and a height of 110 µm using a single-grain diamond dresser, and precise shaping with a CNC machine in the process of dressing the grinding wheel (Table 1). The inner layer has a cylindrical shape.

### 2.1. Measurement Methodology

During the research, the process of surface grinding with intermittent feed was carried out using a CNC SGP250 grinder (manufactured by FAS, Głowno, Poland). During a single pass, a width of the material layer that was removed corresponded to the value of the lateral feed. The ground elements were cuboids with dimensions of 80 mm (L) × 13 mm (W) × 20 mm (H). The grinding process was applied to the Ti-6Al-4V titanium alloy, for which the main alloying elements were 6% of Al and approx. 4% of vanadium (Table 1). The grinding was preceded by shaping and dressing the active surface of the tool, followed by one preliminary machining pass to stabilize its active surface. The parameters of the grinding and shaping processes were constant during all of the tests (Table 1). M = 2 samples were ground with a conventional and aggregate tool.

The measurement of the grinding wheel’s active topography was performed by optical methods (Table 2) using the LEXT 4000 OLS confocal microscope (manufactured by Olympus, Tokyo, Japan) and the Olympus ×20 WD 0.4 lenses. The elementary measurement area was 646 μm × 646 μm, and the magnification ×428. The total test area for one sample was determined according to [20,21], assuming a size of 2972 μm × 2972 μm. For each wheel, three areas were measured, obtained by stitching 25 elementary measurement areas.

**Figure 2:**
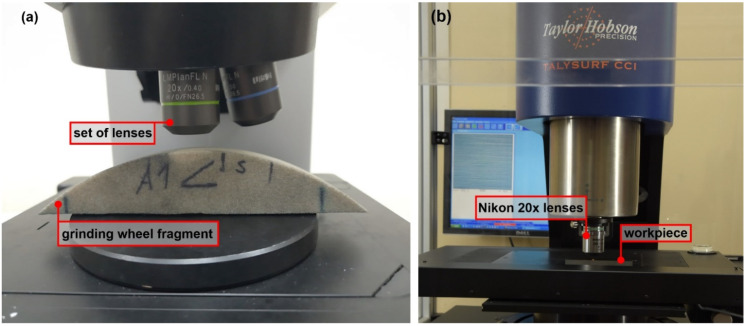
Measurement set- up: (**a**) Olympus OLS LEXT 4000 microscope and (**b**) measurement set-up with Taylor Hobson CCI 6000 and Nikon 20× WD 4.2 lens.

**Figure 3:**
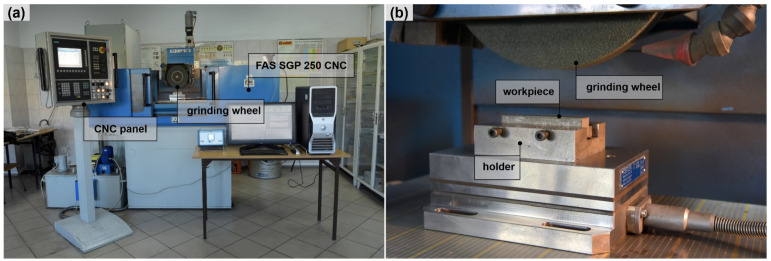
Grinding set-up with FAS CNC grinder; (**a**) CNC machine; (**b**) workpiece holder set-up.

The optical measurement set—up was presented in the Figure 2. The grinding set—up was presented in Figure 3.

The roughness of the ground surfaces was measured with the use of a Talysurf CCI6000 interference profilometer (manufactured by Taylor Hobson, Leciester, Great Britain) and a Nikon lenses with a ×20 magnification, enabling an area with a measurement of 0.899 mm × 0.899 mm (Table 2). The surface of the ground samples was measured in 30 randomly selected places. In each of the selected areas, the measurements were carried out twice and the results were the average.

### 2.2. Methodology of the Surface Roughness Assessment

The analysis of the tools active topography was carried out in the range from the highest surface ordinate to the Δ value, determined according to the following equation:

(1) $$\Delta =Sp-S5p+a_{e},\;\mu m$$

where *Sp* is the maximum peak height (μm), *S*5*p* is the five-point peak height (μm), and *a_e_* is the feed (μm). The reason for the application of these parameter is that the active area of the grinding wheel, in the range of the *Sz* value, should not be entirely involved in the material removal process during the single pass. Thus, because of the need for an estimation of the cutting depth, in order for a proper assessment of the active islands, the level of cutting plane on the measured data could not be obtained by extraction of the cutting depth (grinding process set up) from the top of the peak height. The reason is that the *Sp* value determined on the basis of only one ordinate value exposures the high variability caused by small changes in the active surface of the abrasive tool. A single high point, like a measurement noise, in the measured topography will change the value of this parameter. The *S*5*p* value is determined from the motives using the watershed algorithm and the Wolf pruning [21], and is less variable due to isolated high peaks. 

**Figure 4:**
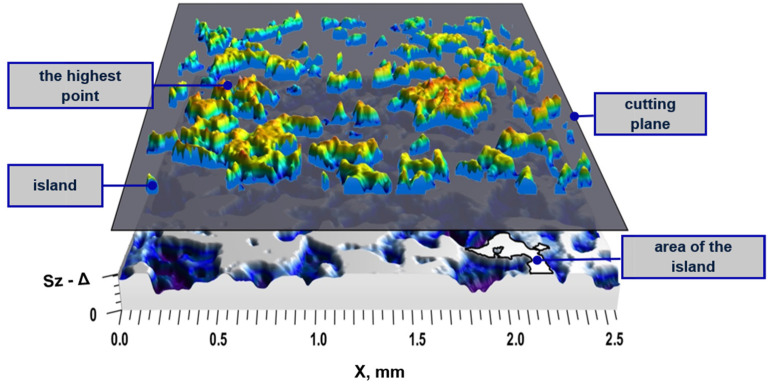
Graphical interpretation of the surface topography intersection at the Δ level.

Parameters describing the tool’s topography were determined from the maximum surface ordinate to the Δ (Figure 4). By dividing this range into 100 layers, the number of the active regions (*Nw*; number of islands), their average area (*Aw*), volume (*Vw*), and the change of the shape factor (*Sw*; according to the following equation) were analyzed for each of them, as follows:

(2) $$Sw=\frac{Vw}{Aw},{\;\mu m}^{3}/{\mu m}^{2}$$

In order to determine the machining potential of the active surface of an abrasive tool, an analysis was performed using the Shos parameter [22].The Shos parameter takes into account the height of the peak (grain or abrasive aggregate), its sharpness, and the orientation of the cutting edges relative to the direction of the machining. The higher the parameter value, the greater the machining potential of the grinding wheel.

### 2.3. Methodology of the Surface Feature Variability Assessment after the Grinding Process

An important step in the analysis of surface roughness parameters is the assessment of their differentiation (their average values) in relation to machining with various tools. This assessment was performed with the use of a bootstrap statistical test [23,24]. Samples made of Ti-6Al-4V material were machined with a conventional grinding wheel (hereinafter referred to as *A*) and a multilayer grinding wheel (designated as *B*). The null hypothesis *H*_0_ and the alternative *H_1_* were formulated as follows:

$$H_{0}:\;\mu_{r\left\{A\right\}}=\mu_{r\left\{B\right\}}$$

$$H_{1}:\;\mu_{r\left\{A\right\}}\neq \mu_{r\left\{B\right\}}$$

where $\mu_{r\left\{A\right\}}$ is the average value of the *p* roughness parameter of the surface ground with grinding wheel *A*, and $\mu_{r\left\{B\right\}}$ is the average value of the *p* roughness parameter of the surface ground with grinding wheel *B*. The distribution of the test statistic values *z* * (assuming *H*_0_) was determined for *R* = 10,000 bootstrap samples $x_{p\left\{A\right\}}^{*}=\left[x_{p{\left\{A\right\}}_{1}}^{*},x_{p{\left\{A\right\}}_{2}}^{*},\ldots ,x_{p{\left\{A\right\}}_{n_{1}}}^{*}\right]$ and $x_{p\left\{B\right\}}^{*}=\left[x_{p{\left\{B\right\}}_{1}}^{*},x_{p{\left\{B\right\}}_{2}}^{*},\ldots ,x_{p{\left\{B\right\}}_{n_{2}}}^{*}\right]$:

(3) $$z^{*}=\frac{{\bar{x}}_{p\left\{A\right\}}^{*}-{\bar{x}}_{p\left\{B\right\}}^{*}-\left({\bar{x}}_{p\left\{A\right\}}-{\bar{x}}_{p\left\{B\right\}}\right)}{\sqrt{\frac{s_{p\left\{A\right\}}^{*2}}{n_{1}}+\frac{s_{p\left\{B\right\}}^{*2}}{n_{2}}}}$$

where ${\bar{x}}_{p\left\{A\right\}}^{*}$, $s_{p\left\{A\right\}}^{*2}$ is the mean value and variance of the *p* roughness parameter of the surfaces ground with tool *A* determined from the bootstrap sample with the size *n*_1_, and ${\bar{x}}_{p\left\{B\right\}}^{*}$, $s_{p\left\{B\right\}}^{*2}$–the mean value and variance of the *p* roughness parameter of the surfaces ground with tool *B* determined from the bootstrap sample with the size *n*_2_.

The bootstrap values $x_{p\left\{A\right\}}^{*}$ i $x_{p\left\{B\right\}}^{*}$ were obtained by resampling the values of the parameters $x_{p\left\{A\right\}}$ and $x_{p\left\{B\right\}}$. The credibility of the *H*_0_ hypothesis was assessed using a *p*-value. This allowed for the assessment of the *H*_0_ hypothesis for any significance level α. If *p*-value > α, it is false to reject the *H*_0_ hypothesis.

The *p*-value was approximated using the following relationship [24]:

(4) $$p=\frac{1+\#\left\{z^{*2}\geq z_{\mathrm{obs}}^{2}\right\}}{R+1}$$

where #{*S*} is the cardinality of *S*.

The observed value of the test statistics *z_obs_* was calculated using the following equation:

(5) $$z_{obs}=\frac{{\bar{x}}_{p\left\{A\right\}}-{\bar{x}}_{p\left\{B\right\}}}{\sqrt{\frac{\sigma_{p\left\{A\right\}}^{2}}{n_{1}}+\frac{\sigma_{p\left\{B\right\}}^{2}}{n_{2}}}}$$

## 3. Results and Discussion

### 3.1. Analysis of the Grinding Wheel Topography

**Figure 5:**
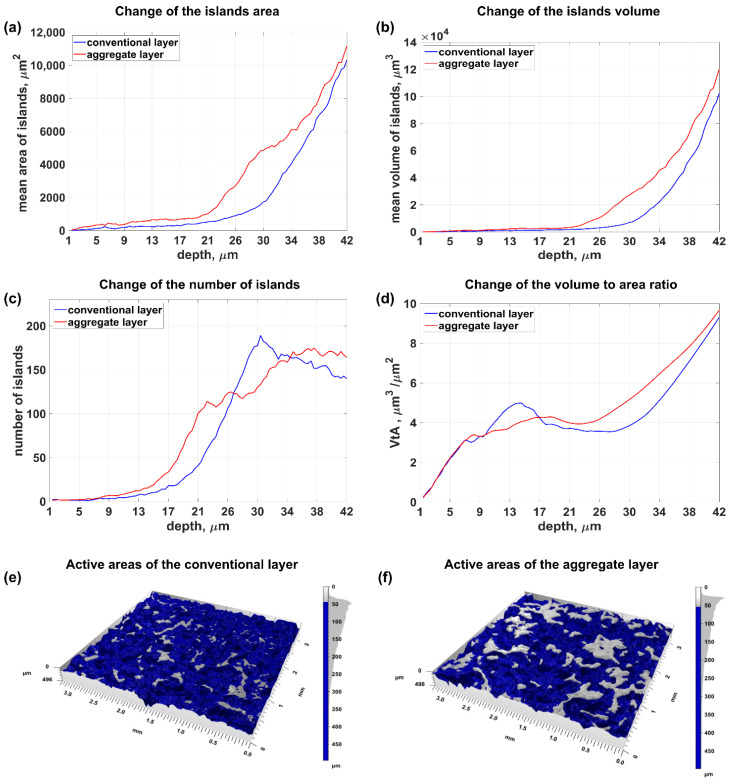
Change in the value of the grinding wheel topography assessment parameters with an increase in the distance from the highest ordinate: (**a**) mean island area, (**b**) mean island volume, (**c**) number of islands, (**d**) shape factor Sw, and (**e**,**f**) visualization of the grinding wheel topography.

Figure 5 shows the change in the value of the parameters for evaluating the active surface of a conventional grinding wheel and a layered grinding wheel. It was observed that the larger volume and area of the islands occurred in the layer with the addition of abrasive aggregates (Figure 5a,b), which resulted from the larger size of the abrasive aggregates themselves in relation to the base abrasive grains. It should be noted that the number of islands, at a depth of up to 25 µm, was greater at the abrasive aggregates layer (Figure 5c). The aggregates are characterized by a large number of small peaks, as they consist of smaller grains. Those peaks are visible as separated islands up to 25 um of depth. As the distance from the highest elevation increases (from 25 µm to 34 µm), the number of islands in the layer with abrasive aggregates is smaller than in the conventional layers. With increasing depth, the islands previously recognized as the areas responsible for the peaks (cutting blades) of the abrasive grains included in the abrasive aggregates merge into common areas. The effect of merging reduced the increase in the number of active regions (observed in the range from 21 µm to 34 µm) while increasing their summary area (Figure 5a).

As the distance from the highest surface ordinate increased, successive grains and abrasive aggregates were exposed. The number of islands increased and then stabilized when new grains were discovered, and the peaks (cutting blades) in the abrasive aggregates were combined into one area of impact (range from 30 µm to 42 µm—Figure 5c).

The shape of the abrasive grains also affected the efficiency of the material removal process. The *Sw* shape factor value analysis showed that the volume of islands related to their area was higher in the layer with abrasive aggregates (range from 17 µm to 42 µm). The value of the *Sw* coefficient, at a depth up to 17 µm, was subject to high uncertainty because the number of active areas in this range was small (about ten islands with a small total area).

**Figure 6:**
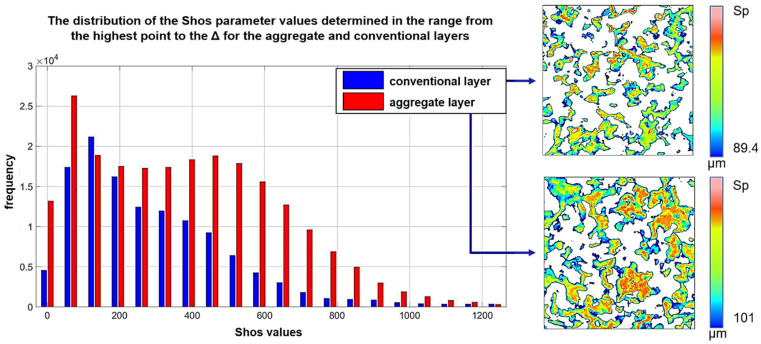
Shos values’ frequency for the aggregate and conventional layers of new abrasive tools in the height range from Sp to Δ.

A detailed analysis of the island’s elevation, sharpness, and directivity is possible using the Shos parameter [22] (Figure 6). Its value increases with: (i) an increase in the elevation of the active area; (ii) an increase in the difference between the elevation of the vertex and the adjacent area (sharpness), and (iii) a decrease in the difference in elevation between the vertex and adjacent areas perpendicular to the machining direction (cutting edge width). The distribution of Shos values was more even for the aggregate layer. Its average value was 1.2 × 10^4^ and was 40% higher than the Shos value for the conventional layer. This confirms the more advantageous geometry of active surfaces in aggregate layers, affecting the efficiency of the material removal process.

### 3.2. Ground Surface Roughness Analysis

**Figure 7:**
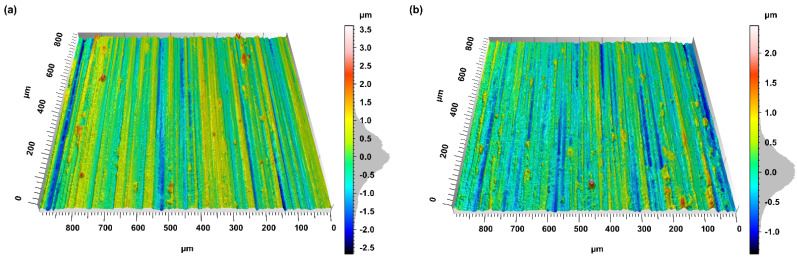
Topographies of the exemplary ground surfaces: (**a**) surface machined with conventional grinding wheel and (**b**) surface machined with a multi-layer grinding wheel.

The impact of abrasive tools on the quality of the machining process was assessed on the basis of the analysis of ground topography measurements. Figure 7 shows exemplary topographies of the machined surfaces. Table 3 presents the summary of the roughness parameter mean values and the results of the bootstrap test for the statistical hypothesis determining the significance of changes in the mean value of the roughness parameters. 

As a result of applying the layer with the addition of abrasive aggregates, the value of the *Ssk* parameter, defining the skewness of the surface ordinates, changed significantly. In the case of surfaces ground with a conventional grinding wheel, the skewness was clearly positive (*Ssk* = 0.78), while in the case of surfaces ground with a layered grinding wheel with abrasive aggregates, the ordinate distribution did not indicate asymmetry (*Ssk* = 0.03).

This indicates the potential of the middle layer to smooth the surface and reduce the proportion of surface elevation. This was confirmed by the analysis of roughness parameters related to the surface elevation analysis, and was determined on the basis of the Abbott−Firestone curve. The value of the *Spk* parameter defining the range of ordinates corresponding to the surface elevations decreased by 31% (from 0.52 µm to 0.36 µm). This indicates a significant reduction of the surface elevations, most of which (about 66%) concerned significant surface elevations and not extreme elevations with a small material ratio (up to 2.5% of the material ratio-*Sxp* parameter).

The elevations of the surfaces machined with the modified grinding wheels were also characterized by a smaller area (*Sha* value lower by 12%) and volume (*Shv* value lower by 34%) in relation to the surface elevations obtained as a result of grinding with a conventional grinding wheel. Taking into account the ratio of the volume of the elevations (*Shv*) to their surface (*Sha*), it can be noticed that in the case of surfaces grinded with aggregate grinding wheels, the peaks of the surfaces were less sharp (flatter). This increased the bearing capacity of the surface, and helped to transfer the load.

The addition of abrasive aggregates, which have a greater ability to micro-sharpen and contain smaller grains, removed the surface elevation resulting from machining with a conventional layer (also removing elevation resulting from material redeposition and side ridges). The use of grains smaller than the basic grains in the abrasive aggregates did not result in new deeper cracks than those formed after grinding with a conventional layer. In turn, the advantageous design of the cutting blades in relation to the machining direction (higher value of the *Shos* parameter) resulted in more effective removal of the machined material (reduction of the roughness parameters *Sa* and *Sq*).

**Figure 8:**
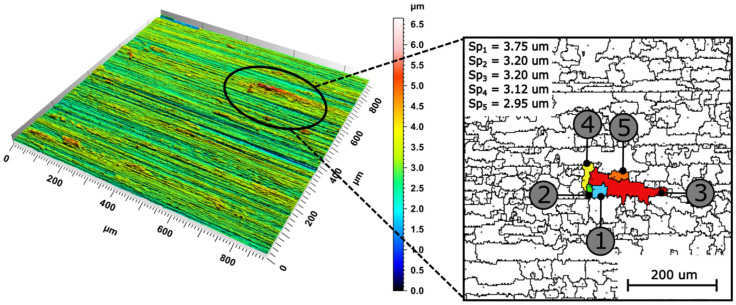
Illustration of the features of five surface elevations (parameter S5p) after Wolf pruning (5% Sz).

In the results of the analysis of the ground surfaces roughness, for both types of abrasive tools, it should also be noted that parameters *Sz*, *Sp*, and *Sv* were insensitive to changes occurring on the machined surface. These parameters were sensitive to small changes on the surface (they are determined on the basis of one or two values from over a million ordinates of the surface obtained as a result of the measurement) and were characterized by a high variability. No changes were also observed in the case of the parameter *S*5*p* (as well as *S*10*z*), determined on the basis of the height of the five highest peaks. A detailed analysis of the insensitivity of the parameters of the features to changes in the topography of the analyzed surfaces was performed (Figure 8).

The feature parameters were determined based on the watershed. The use of a watershed on surfaces after grinding, measured by optical methods, led to significant segmentation. The method of preventing over-segmentation is to merge separate areas (peaks or valleys) connected by a common pass (saddle point). The ISO 25178-2: 2012 [21] standard assumed the joining of separate areas when the difference in the height of areas was less than 5% of the *Sz* parameter value (using the Wolf pruning method). In the case of the surface shown in Figure 8, this resulted in reducing the number of separate areas from 46,000 (without pruning) to 2469 (after pruning). Nevertheless, the presence of high peaks on the analyzed surfaces (caused, among others, by the adhered material and significant plastic deformations due to fractures/pull-out of the abrasive grains) caused the formation of areas in which all surface elevations were concentrated, on the basis of which the determination of the parameter *S*5*p* is made. As a result, a small part of the analyzed area determined the value of the parameter. This contributed to the high variability of this parameter, which, in the case of the analyzed surfaces, caused its inability to differentiate surface features.

## 4. Conclusions

This paper describes the results of research on the grinding wheels’ active surfaces. Conventional and multi-layered tools were tested. Based on the analyses, the following was found:

1. The addition of abrasive aggregates increases the size of the active areas of the grinding wheels. These areas are characterized by favorable geometrical features related, inter alia, to the increased width of the cutting edges compared to the active areas present on the surface of the conventional grinding wheel.
2. The assessment of the grinding wheel’s cutting ability was carried out using the *Shos* parameter. It allows for assessing the elevation of the active surfaces, their sharpness, and the orientation of the cutting edges in relation to the cutting direction. The *Shos* value for the layer with abrasive aggregates is 40% higher than for the layer with conventional abrasive grains;
3. The use of bootstrap for the statistical hypothesis tests makes it possible to evaluate the differentiation of the ground surface features as a result of mean roughness parameters values. Those analyses take into account the actual form of the probability distribution of those parameters. They also express the irregularity of the surface roughness parameter values as a result of the grinding process variability;
4. The modification of the grinding wheels as an application of new middle layer containing the addition of abrasive aggregates increases the tool’s ability to smooth the machined surface. In the case of surfaces ground with multilayer grinding wheels, more favorable values of roughness parameters were observed for the group of amplitude parameters (*Sa*, *Sq*, and *Ssk*), functional parameters (*Spk*, *Vmp*, and *Sxp*), and feature parameters (*Sha* and *Shv*);
5. The effects of the application of an intermediate layer with the participation of abrasive aggregates affects the load-bearing capacity of the machined surfaces. The use of abrasive aggregates, with an impact surface larger than the base grains, results in the formation of a topography characterized by a smaller area (Sha value lower by 12%) and a smaller volume of peaks (*Shv* value lower by 34%) compared to the surfaces obtained as a result of grinding with a conventional grinding wheel.

## Figures and Tables

**Table 1:** Grinding set up.

**Process Parameters**	
grinding method	reciprocating grinding
workpiece material	Ti-6Al-4V
grinding wheel speed	v_s_ = 18 m/s
lateral feed	a_p_ = 1 mm/stroke
feed rate	v_w_ = 4 m/min
depth of cut	a_e_ = 0.1 mm
dresser	single-point diamond dresser
dressing depth (ad)	a_d_ = 0.05 mm
dressing speed (vd)	v_d_ = 5 mm/s
grinding condition	wet grinding
coolant	EMU 12 in 5% water solution
coolant preassure	7 bar
coolant flow rate	20 L/min
**Ti-6Al-4V Workpiece Properties**	
Average tensile strength MPa	895
Yield point MPa	825
Young’s Modulus GPa	110
Thermal conductivity W/(m·K)	6.7 (20 °C)
Density g/cm^3^	4.43

**Table 2:** Parameters of the tool active area measurements.

**Grinding Wheel Topography Measurement**	
Equipment	Confocal microscope LEXT OLS4000 with Anti Vibrant AV1 table
Lenses	Olympus 20×, WD = 0.4
Magnification	×428
Elementary measurement area	646 μm × 646 μm for Olympus × 20 lense, with numerical magnification x1
Applied stitching	5 × 5 cells with 10% overlap
Number of areas measured	3 × (2972 μm × 2972 μm) for each tool
**Specimen Topography Measurement**	
Equipment	Interference profilometer Taylor Hobson CCI 6000
Lenses	Nikon × 20/0.40DI WD 4.7
Magnification	×428
Elementary measurement area	899 μm × 899 μm for Olympus ×20 lense, with numerical magnification ×1
Number of areas measured	60 × (899 μm × 899 μm)

**Table 3:** Average values of the roughness parameters [21] of the ground surfaces and the results of the bootstrap test of the statistical significance of their differences.

Parameter	Conventional Tool	Multilayer Tool	Unit	*p*-Value	Statistically Significant Difference
Amplitude parameters					
*Sq*	0.37 ± 0.009	0.33 ± 0.006	µm	2.62 × 10^−4^	yes
*Ssk*	0.78 ± 0.09	0.03 ± 0.08		9.99 × 10^−6^	yes
*Sku*	7.5 ± 0.4	6.2 ± 0.5		6.31 × 10^−2^	no
*Sp*	2.6 ± 0.1	2.5 ± 0.1	µm	3.84 × 10^−1^	no
*Sv*	2.0 ± 0.1	1.8 ± 0.1	µm	2.86 × 10^−1^	no
*Sz*	4.6 ± 0.2	4.3 ± 0.1	µm	1.94 × 10^−1^	no
*Sa*	0.27 ± 0.006	0.25 ± 0.005	µm	3.01 × 10^−2^	yes
Functional parameters					
*Smr*	0.9 ± 0.1	0.9 ± 0.6	%	8.91 × 10^−1^	no
*Smc*	0.42 ± 0.01	0.39 ± 0.01	µm	2.00 × 10^−2^	yes
*Sxp*	0.62 ± 0.02	0.68 ± 0.02	µm	2.20 × 10^−2^	yes
Spatial parameters					
*Sal*	21.8 ± 0.7	14.4 ± 0.3	µm	9.89 × 10^−6^	yes
*Str*	0.0973 ± 0.0060	0.0334 ± 0.0009		9.99 × 10^−6^	yes
Hybrid parameters					
*Sdq*	0.065 ± 0.001	0.066 ± 0.001		2.74 × 10^−1^	no
*Sdr*	0.21 ± 0.01	0.22 ± 0.01	%	2.59 × 10^−1^	no
Volume parameters					
*Vv*	0.46 ± 0.01	0.41 ± 0.01	µm³/µm²	2.30 × 10^−3^	yes
*Vmp*	0.033 ± 0.001	0.019 ± 0.001	µm³/µm²	9.99 × 10^−6^	yes
*Vmc*	0.27 ± 0.01	0.27 ± 0.01	µm³/µm²	6.69 × 10^−1^	no
*Vvc*	0.42 ± 0.01	0.36 ± 0.01	µm³/µm²	8.29 × 10^−6^	yes
*Vvv*	0.0384 ± 0.0013	0.0421 ± 0.0011	µm³/µm²	3.29 × 10^−2^	yes
Features parameters					
*Spd*	0.00025 ± 0.00001	0.00030 ± 0.00001	1/µm²	7.70 × 10^−3^	yes
*Spc*	0.045 ± 0.001	0.049 ± 0.000	1/µm	1.20 × 10^−4^	yes
*S10z*	2.9 ± 0.1	2.8 ± 0.1	µm	1.71 × 10^−1^	no
*S5p*	1.76 ± 0.06	1.75 ± 0.04	µm	9.63 × 10^−1^	no
*S5v*	1.18 ± 0.07	1.03 ± 0.04	µm	4.88 × 10^−2^	yes
*Sda*	3463 ± 155	2844 ± 136	µm²	3.80 × 10^−3^	yes
*Sha*	3475 ± 143	3060 ± 138	µm²	3.10 × 10^−2^	yes
*Sdv*	104 ± 9	71 ± 6	µm³	3.10 × 10^−3^	yes
*Shv*	129 ± 10	83 ± 6	µm³	4.90 × 10^−4^	yes
Functional parameters					
*Sk*	0.67 ± 0.01	0.69 ± 0.01	µm	3.07 × 10^−1^	no
*Spk*	0.52 ± 0.02	0.36 ± 0.01	µm	9.99 × 10^−6^	yes
*Svk*	0.33 ± 0.02	0.35 ± 0.01	µm	4.23 × 10^−1^	no
*Smr1*	11.8 ± 0.3	9.9 ± 0.2	%	9.99 × 10^−6^	yes
*Smr2*	89.3 ± 0.2	88.3 ± 0.2	%	4.90 × 10^−3^	yes

## Data Availability

Data sharing is not applicable to this article.
